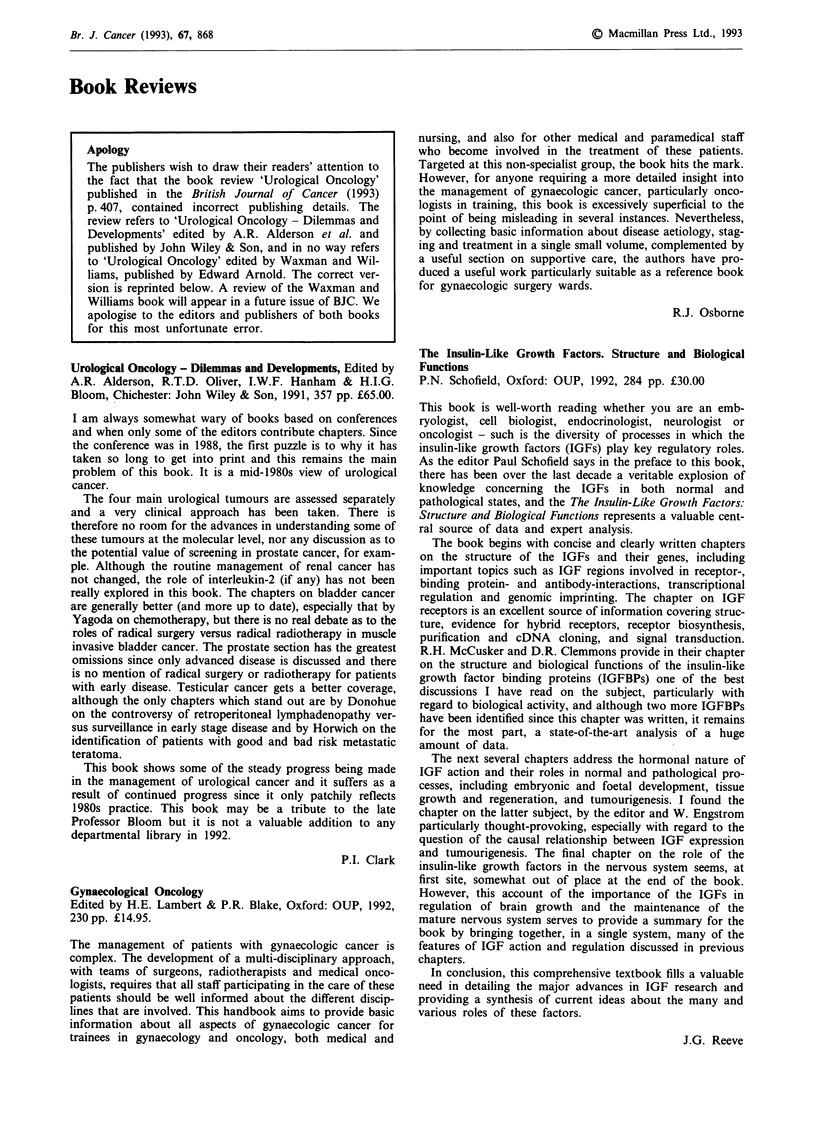# The Insullin-Like Growth Factors. Structure and Biological Functions

**Published:** 1993-04

**Authors:** J.G. Reeve


**The Insulin-Like Growth Factors. Structure and Biological Functions**
P.N. Schofield, Oxford: OUP, 1992, 284 pp. £30.00

This book is well-worth reading whether you are an embryologist, cell biologist, endocrinologist, neurologist or oncologist – such is the diversity of processes in which the insulin-like growth factors (IGFs) play key regulatory roles. As the editor Paul Schofield says in the preface to this book, there has been over the last decade a veritable explosion of knowledge concerning the IGFs in both normal and pathological states, and the *The Insulin-Like Growth Factors: Structure and Biological Functions* represents a valuable central source of data and expert analysis.

The book begins with concise and clearly written chapters on the structure of the IGFs and their genes, including important topics such as IGF regions involved in receptor-, binding protein- and antibody-interactions, transcriptional regulation and genomic imprinting. The chapter on IGF receptors is an excellent source of information covering structure, evidence for hybrid receptors, receptor biosynthesis, purification and cDNA cloning, and signal transduction. R.H. McCusker and D.R. Clemmons provide in their chapter on the structure and biological functions of the insulin-like growth factor binding proteins (IGFBPs) one of the best discussions I have read on the subject, particularly with regard to biological activity, and although two more IGFBPs have been identified since this chapter was written, it remains for the most part, a state-of-the-art analysis of a huge amount of data.

The next several chapters address the hormonal nature of IGF action and their roles in normal and pathological processes, including embryonic and foetal development, tissue growth and regeneration, and tumourigenesis. I found the chapter on the latter subject, by the editor and W. Engstrom particularly thought-provoking, especially with regard to the question of the causal relationship between IGF expression and tumourigenesis. The final chapter on the role of the insulin-like growth factors in the nervous system seems, at first site, somewhat out of place at the end of the book. However, this account of the importance of the IGFs in regulation of brain growth and the maintenance of the mature nervous system serves to provide a summary for the book by bringing together, in a single system, many of the features of IGF action and regulation discussed in previous chapters.

In conclusion, this comprehensive textbook fills a valuable need in detailing the major advances in IGF research and providing a synthesis of current ideas about the many and various roles of these factors.

J.G. Reeve